# Hyperspectral Imaging for Mycotoxin Monitoring in Food: A Comprehensive Review of Technologies, Algorithms, and Applications

**DOI:** 10.3390/toxins18090396

**Published:** 2026-09-16

**Authors:** Guangyao Ying, Maofa Zeng, Liang Hong, Zhaokui Li, Jun Li, Huili Xia

**Affiliations:** 1 Taizhou Institute for Food and Drug Control, Taizhou 318000, China; tzsyygy@126.com (G.Y.);; 2 Key Laboratory for Quality Safety and Quality Improvement of Characteristic Agricultural Products in Taizhou City, Taizhou 318000, China; 3 Taizhou Key Laboratory of Model Animal and Preclinical Pharmaceutical Research, Taizhou 318000, China; 4 Taizhou Municipal Key Laboratory of Digital and Intelligent Testing and Quality Evaluation of Traditional Chinese Medicine, Taizhou 318000, China; 5 Taizhou Municipal Key Laboratory of Drug Composition and Adulteration Identification Technology, Taizhou 318000, China

**Keywords:** hyperspectral imaging, mycotoxins, machine learning, deep learning, food safety, non-destructive detection, spectral analysis

## Abstract

Mycotoxin contamination represents one of the most pressing food safety challenges worldwide, with millions of tons of agricultural commodities affected annually. Conventional detection methods, while accurate, are labor-intensive, destructive, and unsuitable for large-scale screening. Hyperspectral imaging (HSI) has emerged as a transformative non-destructive analytical technique capable of simultaneously capturing spatial and spectral information across hundreds of contiguous wavelengths. This review critically evaluates the current state of HSI technology for mycotoxin detection in food products, covering the physical principles underlying spectral–mycotoxin interactions, systematic applications across major mycotoxin classes (aflatoxins, deoxynivalenol, ochratoxin A, fumonisins, and zearalenone), and the integration of machine learning and deep learning algorithms for spectral data analysis. The review reveals that while Vis-NIR (400–1000 nm) and SWIR (1000–2500 nm) HSI systems have achieved classification accuracies exceeding 90% for several mycotoxin–matrix combinations, fundamental challenges persist in model transferability, direct quantification at regulatory thresholds, and scalability for industrial deployment. Recent advances in transformer architectures, transfer learning, interpretable deep learning, and portable multispectral systems demonstrate encouraging progress toward practical implementation. This review concludes by identifying critical research gaps and proposing strategic directions for translating HSI-based mycotoxin detection from laboratory proof-of-concept to routine industrial application.

## 1. Introduction

Mycotoxins, toxic secondary metabolites produced by fungal genera including *Aspergillus*, *Fusarium*, and *Penicillium*, have been estimated by the Food and Agriculture Organization (FAO) to contaminate approximately 25% of global crop production annually, although more recent analyses suggest substantially higher occurrence rates when considering detectable contamination levels [1], and pose severe health risks to humans and livestock [2,3,4]. Among the more than 400 identified mycotoxins, aflatoxins (AFs), deoxynivalenol (DON), ochratoxin A (OTA), fumonisins (FBs), and zearalenone (ZEN) are subject to regulatory limits in many countries and regions, although the scope of regulated toxins and maximum permitted concentrations vary considerably across jurisdictions due to their carcinogenic, nephrotoxic, and immunosuppressive properties [2,3]. The International Agency for Research on Cancer classifies aflatoxins as Group 1 carcinogens, OTA and fumonisins as Group 2B, and DON and ZEN as Group 3 carcinogens [2]. Given the heterogeneity of contamination distribution even within individual grain lots, conventional analytical methods such as high-performance liquid chromatography (HPLC), enzyme-linked immunosorbent assay (ELISA), and liquid chromatography-tandem mass spectrometry (LC-MS/MS) face inherent limitations in sampling representativeness despite their analytical precision [2,5].

HSI technology has attracted substantial research interest as a rapid, non-destructive alternative that integrates the spatial resolution of computer vision with the chemical specificity of spectroscopy [4,5,6]. Each pixel in a hyperspectral data cube contains a complete reflectance, absorbance, or fluorescence spectrum, enabling simultaneous assessment of contamination extent and chemical composition [7,8,9]. The technology operates across multiple spectral regions: visible/near-infrared (Vis-NIR, 400–1000 nm), near-infrared (NIR, 900–1700 nm), short-wave infrared (SWIR, 1000–2500 nm), and fluorescence HSI [10,11,12]. By probing vibrational overtones and combination bands of fundamental molecular bonds (C-H, O-H, N-H), HSI captures spectral signatures correlated with mycotoxin presence and fungal metabolic activity [3,6].

The convergence of HSI with artificial intelligence, particularly machine learning (ML) and deep learning (DL) algorithms, has fundamentally accelerated the field’s development [7,13,14]. These computational methods address the high dimensionality, collinearity, and spectral noise inherent in hyperspectral data while extracting latent patterns that distinguish contaminated from uncontaminated samples [2,15,16]. Despite these advances, significant technical barriers remain, including model robustness across diverse sample origins, the indirect nature of mycotoxin detection through correlated spectral changes, and the translation of laboratory systems to high-throughput industrial environments [2,8,17].

This review provides a systematic and critical analysis of HSI applications for mycotoxin monitoring in food products. The scope encompasses (1) the physical and technical foundations of HSI systems for mycotoxin detection; (2) detailed evaluation of applications across major mycotoxin classes; (3) comparative assessment of ML and DL algorithms for spectral analysis; (4) advances in spectral preprocessing and feature selection; (5) fluorescence imaging and Raman spectroscopy as complementary modalities; (6) industrial implementation challenges and portable system development; and (7) identification of research gaps and future directions. Throughout, this review emphasizes critical evaluation over descriptive summary, examining methodological limitations, contradictory findings, and the mechanistic basis of detection capabilities across the literature.

The literature search for this review was conducted using the Web of Science database, covering publications from 2020 to 2026. The search strategy employed combinations of the following keywords: “hyperspectral imaging,” “mycotoxin,” and “food.” Priority was given to peer-reviewed journal articles reporting original HSI-based studies on mycotoxin detection and relevant review articles providing contextual background.

While several reviews have examined aspects of hyperspectral imaging for mycotoxin detection [2,9,16,17,18], this review offers several distinctive contributions that advance the field beyond existing literature. First, this is the first review to systematically integrate and critically evaluate the application of Transformer architectures and interpretable deep learning approaches, particularly SHAP (SHapley Additive exPlanations) analysis, in the context of HSI-based mycotoxin detection, moving beyond conventional CNN-based methods to assess the emerging role of attention mechanisms and model explainability in building detection confidence [19,20,21]. Second, this review is the first to dedicate a comprehensive section to comparing fluorescence HSI and Raman HSI as complementary spectroscopic modalities to reflectance-based HSI, examining how these approaches can address the fundamental limitation of indirect mycotoxin detection through direct molecular signatures [22,23]. Third, the review incorporates the most recent advances published through 2026, including snapshot HSI for real-time industrial screening [24], transfer learning strategies for cross-domain model adaptation [25], and data augmentation techniques for overcoming training data limitations [26]. Fourth, and most importantly, this review establishes a complete analytical logic chain from the physical basis of indirect detection, through algorithmic feature extraction and model development, to the regulatory compliance requirements essential for practical deployment, providing a holistic framework that connects fundamental detection mechanisms with the practical requirements for real-world food safety implementation.

## 2. Principles and Technical Foundations

**Figure 1:**
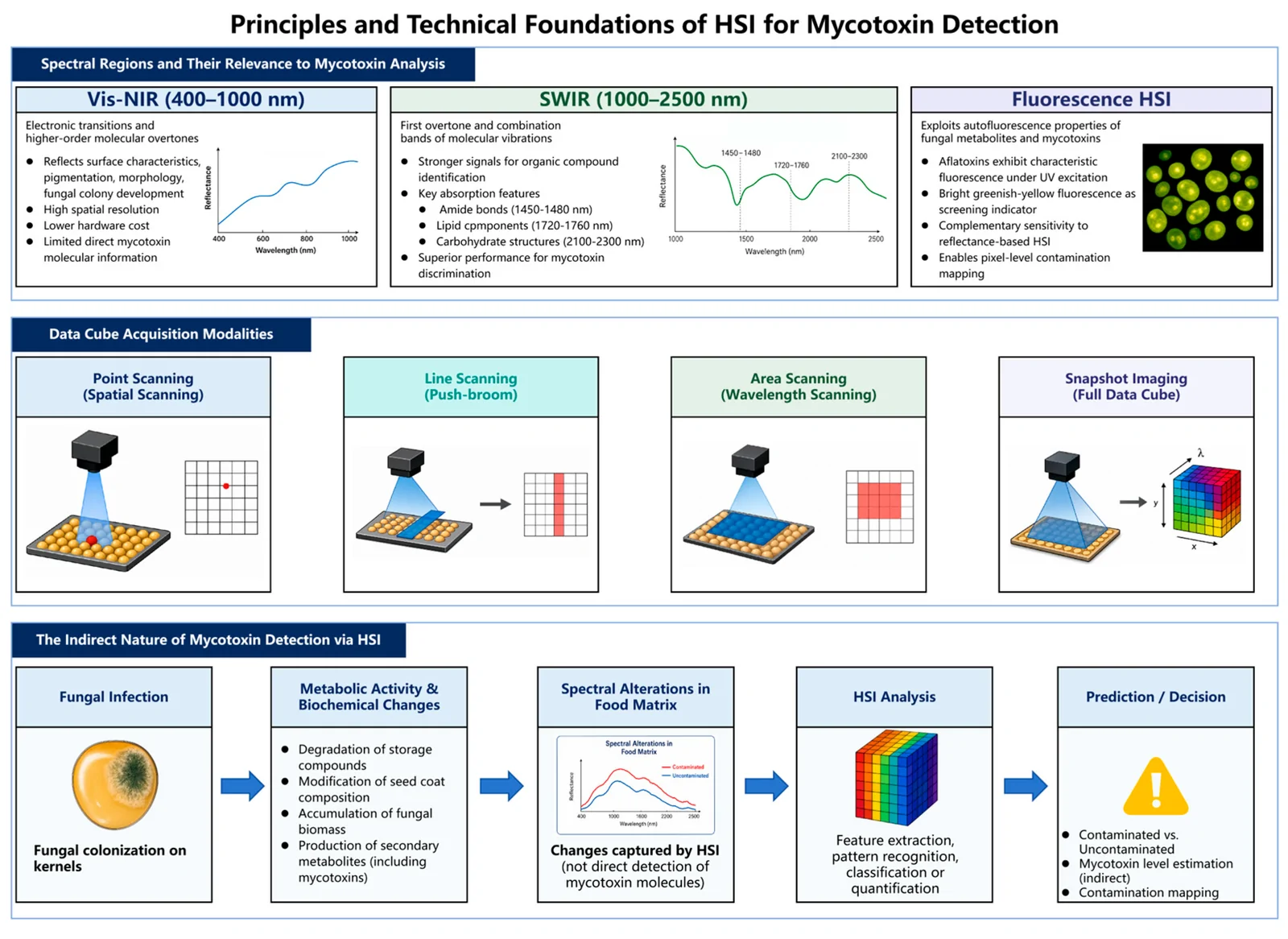
Principles and Technical Foundations of HSI for Mycotoxin Detection.

The effective application of hyperspectral imaging to mycotoxin detection requires a solid understanding of the underlying physical principles, technical configurations, and fundamental characteristics that distinguish this approach from conventional analytical methods. The interaction between electromagnetic radiation and food matrices involves complex physical and chemical processes that determine which spectral regions are most informative for mycotoxin-related analysis, how hyperspectral data are acquired across different instrumental configurations, and why the detection mechanism is inherently indirect. This section establishes the technical and conceptual foundations necessary for critically evaluating the HSI-based mycotoxin detection literature, covering the spectral regions most relevant to mycotoxin analysis, the principal data acquisition modalities and their trade-offs, and the fundamental nature of indirect detection that shapes both the capabilities and limitations of this technology. The key principles and technical configurations are summarized in Figure 1.

### 2.1. Spectral Regions and Their Relevance to Mycotoxin Analysis

The selection of spectral region fundamentally determines the detection capability of HSI for mycotoxin analysis. Vis-NIR systems (400–1000 nm) detect electronic transitions and higher-order molecular overtones, offering high spatial resolution and lower hardware costs [12,18]. However, spectral information in this region primarily reflects surface characteristics, pigmentation, morphological changes, and fungal colony development, rather than direct mycotoxin molecular signatures [11,19]. The transition from visible to NIR wavelengths involves a trade-off between spatial resolution and chemical specificity, as longer wavelengths carry richer compositional information but require more sensitive detectors [10,12].

SWIR systems (1000–2500 nm) provide access to first overtone and combination bands of molecular vibrations, delivering substantially stronger signals for organic compound identification [10,11,20]. The SWIR region encompasses characteristic absorption features for fungal metabolites, including amide bonds (1450–1480 nm), lipid components (1720–1760 nm), and carbohydrate structures (2100–2300 nm) that change during fungal infection and mycotoxin biosynthesis [11,20]. Chu et al. demonstrated that SWIR HSI achieved superior discrimination of aflatoxin B1 (AFB1)-contaminated maize kernels compared to Vis-NIR systems, with key wavelengths at 1206, 1380, 1664, 1858, and 2130 nm contributing significantly to classification performance [11]. Kimuli et al. further validated the SWIR advantage, achieving classification accuracies exceeding 90% for AFB1-contaminated maize kernels using partial least squares discriminant analysis (PLS-DA) on SWIR spectral data [20].

Fluorescence HSI exploits the autofluorescence properties of fungal metabolites and mycotoxins [21,22]. Aflatoxins exhibit characteristic fluorescence under UV excitation, with bright greenish-yellow fluorescence (BGYF) historically serving as a screening indicator for contaminated corn kernels [21,22]. The integration of fluorescence spectral data with spatial information enables pixel-level contamination mapping, offering detection sensitivity complementary to reflectance-based HSI [22,23].

### 2.2. Data Acquisition Modalities

Hyperspectral data can be collected through four acquisition configurations: point scanning (spatial scanning), line scanning (push-broom), area scanning (wavelength scanning), and snapshot imaging (full data cube) [6,10]. Push-broom line scanning dominates food and agricultural applications due to its favorable balance between acquisition speed, spectral quality, and spatial resolution [1,6]. This configuration acquires one spatial line per image frame, building the complete data cube through sample translation [1,6]. While offering reliable spectral quality, push-broom systems require mechanical sample movement, limiting throughput for industrial applications [6,17].

Snapshot HSI represents a transformative approach for real-time monitoring, capturing the complete spatial-spectral data cube in a single exposure without mechanical scanning [17,24]. Although currently limited by lower spatial resolution and fewer spectral bands compared to push-broom systems, snapshot technology eliminates the scanning bottleneck and enables deployment on conveyor systems [17]. The recent development of snapshot multispectral imaging with deep learning enhancement demonstrates potential for high-speed contamination screening [24].

### 2.3. The Indirect Nature of Mycotoxin Detection via HSI

The spectral changes captured by HSI correspond to specific physicochemical alterations that occur across multiple wavelength regions during fungal infection and mycotoxin biosynthesis. In the visible range (400–700 nm), degradation of chlorophyll and carotenoids in infected kernels produces detectable reflectance shifts, which are particularly pronounced in the red-edge transition region near 680–750 nm [12,27]. In the near-infrared region, water absorption bands near 970, 1450, and 1940 nm reflect moisture redistribution caused by fungal colonization and metabolic water production [9,28]. Carbohydrate-related absorptions, including C–H and O–H overtones around 1200, 1700–1800, and 2100–2300 nm, are sensitive to starch degradation and the conversion of storage polysaccharides into simpler sugars during fungal growth [12,28,29]. Protein-associated absorptions near 1480 and 2050–2200 nm (amide and peptide bond combinations) reflect both host protein breakdown and the accumulation of fungal enzymes [27,30]. Lipid C–H stretching and combination bands around 1720–1760 and 2300–2350 nm indicate lipid metabolism and membrane disruption during infection [12,29]. Beyond molecular composition, fungal colonization alters tissue microstructure, cell wall degradation, mycelial proliferation, and cavity formation, changing light scattering patterns across the full spectral range [7,9]. Fungal biomass itself contributes diagnostic spectral signals through cell wall components such as ergosterol and chitin [31]. Because these biochemical and structural changes occur concurrently during infection, HSI spectra reflect a composite biological response rather than a toxin-specific molecular signature, which has important implications for model specificity and transferability across different matrices and contamination scenarios [9,28].

A fundamental distinction requires emphasis: conventional HSI does not detect mycotoxin molecules directly at regulatory concentrations. Rather, HSI captures spectral alterations in the food matrix resulting from fungal infection, metabolic activity, and the biochemical changes accompanying mycotoxin biosynthesis [6,8,25]. These alterations include modification of seed coat composition, accumulation of fungal biomass, degradation of storage compounds, and production of secondary metabolites with distinct spectral signatures [6,8,26].

The indirect detection mechanism introduces both advantages and limitations. It enables rapid screening without sample preparation, but the correlation between spectral features and actual mycotoxin concentration depends on the specific biological relationship between fungal growth and toxin production, which varies with environmental conditions, substrate composition, and fungal strain [8,25]. This fundamental constraint explains why many HSI studies report successful classification of contaminated versus uncontaminated samples but encounter greater difficulty in quantitative prediction at low concentration levels [25,27,32].

## 3. HSI Applications in Mycotoxin Detection and Fungal Classification

**Figure 2:**
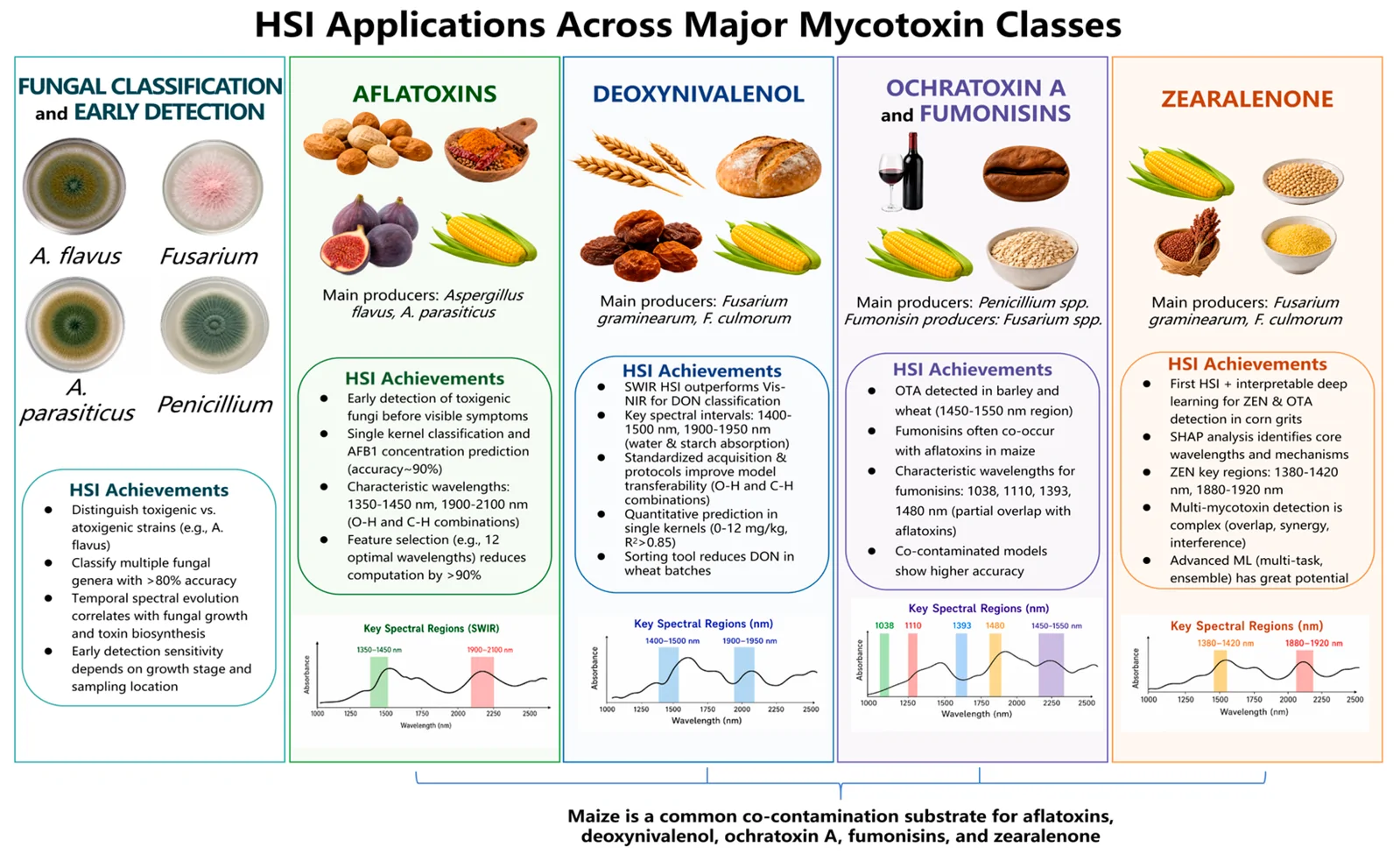
HSI applications across major mycotoxin classes. The figure summarizes detection capabilities, key spectral regions, and representative applications for aflatoxins [2,12,27,29,33,34,35,36], deoxynivalenol (DON) [10,28,30,37,38,39,40,41], ochratoxin A (OTA) and fumonisins [3,17,42,43], zearalenone (ZEN) and multi-mycotoxin contamination [3,16,17,19,26,40,44], and fungal classification and early detection [45,46,47]. Notably, maize serves as a common co-contamination substrate for multiple mycotoxin classes, particularly aflatoxins, fumonisins, and zearalenone, posing challenges for simultaneous detection [3,17].

The application of hyperspectral imaging to mycotoxin detection has been investigated across a diverse range of mycotoxin classes, food matrices, and contamination scenarios. The breadth of this research reflects both the regulatory importance of different mycotoxins and the varying degrees of spectral sensitivity achievable for each toxin–matrix combination. This section systematically reviews HSI applications organized by major mycotoxin classes, examining the detection capabilities, methodological approaches, and limitations reported for each. The analysis reveals that detection performance is strongly influenced by the spectral region employed, the biological relationship between fungal infection and toxin production, and the specific food matrix under investigation. An overview of these applications is provided in Figure 2.

### 3.1. Fungal Classification and Early Detection

Beyond mycotoxin quantification, HSI has been extensively applied to fungal species identification and early detection. Jin et al. classified toxigenic and atoxigenic strains of *A. flavus* using HSI, demonstrating that spectral differences between toxin-producing and non-producing strains enabled classification with accuracies exceeding 80% [45]. Yao et al. differentiated among multiple toxigenic fungal genera (*Fusarium*, *A. flavus*, *Trichoderma*, *A. parasiticus*, and *Penicillium*) using Vis-NIR HSI, establishing the foundation for species-specific detection strategies [46].

Lu et al. evaluated and classified five cereal fungi (*A. parasiticus*, *A. flavus*, *A. glaucus*, *A. niger*, and *Penicillium* spp.) on culture medium using Vis/NIR HSI, demonstrating temporal spectral evolution patterns specific to each species [47]. The classification accuracy improved with incubation time as fungal biomass increased spectral signal intensity, revealing that early detection sensitivity depends critically on the temporal dynamics of fungal growth [47].

### 3.2. Aflatoxins

Aflatoxins, primarily produced by *Aspergillus flavus* and *A. parasiticus*, represent the most extensively studied mycotoxin class in HSI research due to their potent carcinogenicity and regulatory significance [2,12,33]. Del Fiore et al. pioneered HSI-based early detection of toxigenic fungi on maize kernels, demonstrating that hyperspectral analysis could identify fungal colonization before visible symptoms appeared, establishing the foundational concept of pre-symptomatic detection [33].

Subsequent research systematically refined detection capabilities across multiple dimensions. Wang et al. established the feasibility of identifying AFB1 on maize kernel surfaces using SWIR HSI (1000–2500 nm), achieving classification accuracies exceeding 85% through PLS-DA modeling [29]. Chu et al. extended this work to individual kernel analysis, identifying optimal wavelengths through PCA loading analysis and demonstrating that SWIR HSI could distinguish naturally contaminated from uncontaminated maize kernels with high specificity [11]. The identification of characteristic wavelengths, particularly in the 1350–1450 nm and 1900–2100 nm regions corresponding to O-H and C-H combination bands, provided mechanistic insight into the spectral basis of aflatoxin detection [11,20].

The relationship between fungal growth stage and spectral signature evolution has been systematically investigated. Guo et al. evaluated *A. flavus* growth dynamics on maize agar culture medium using Vis/NIR HSI, revealing temporal spectral patterns that correlated with mycelial expansion and AFB1 biosynthesis [33]. Lu et al. advanced this investigation by combining SWIR HSI with synchrotron FTIR microscopy to probe spatio-temporal patterns of *A. flavus* infection, demonstrating that fungal colonization progressed from the germ region outward and that AFB1 accumulation exhibited heterogeneous spatial distribution within individual kernels [48]. These multi-scale analyses established that spectral detection sensitivity depends critically on sampling location relative to fungal colonization patterns.

Single kernel analysis represents a critical advancement for practical application. Chakraborty et al. demonstrated non-destructive classification and prediction of AFB1 concentration in individual maize kernels using Vis-NIR HSI, achieving detection accuracies of approximately 90% through SVM and PLSR modeling [19]. Kim et al. extended single- and multi-mycotoxin detection to ground maize samples, demonstrating that HSI coupled with machine learning could simultaneously detect aflatoxins and fumonisins in co-contaminated samples, addressing the prevalent reality of multi-mycotoxin occurrence [2].

Feature wavelength selection has substantially improved model efficiency. Zhou et al. developed a feature selection approach combining spectral and image information for AFB1 classification, identifying 12 optimal wavelengths that maintained classification accuracy while reducing computational requirements by over 90% [28]. The CARS (competitive adaptive reweighted sampling) algorithm and SPA (successive projections algorithm) have been extensively validated as effective wavelength selection methods for aflatoxin detection models [28,30].

Beyond maize and peanuts, tree nuts represent another significant matrix for aflatoxin contamination. Recent studies have extended HSI applications to almond and pistachio kernels. Kabir et al. investigated SWIR-HSI (1000–2500 nm) combined with deep learning for AFB1 detection in almond kernels, demonstrating that a pre-trained Inception V3 network achieved 84.82% cross-validation accuracy, highlighting the superiority of deep learning approaches for handling the diverse geometries of almond kernels [35]. Williams et al. applied machine learning techniques to hyperspectral images of pistachio nuts, achieving 96.7% accuracy in classifying kernels into three contamination tiers (<8, >160, and >300 μg/kg), demonstrating the potential of HSI for industrial-quality control of pistachios [36].

### 3.3. Deoxynivalenol

DON contamination of wheat and barley represents the most economically significant mycotoxin problem in temperate cereal production systems. DON is primarily produced by *Fusarium graminearum* and *Fusarium culmorum* in wheat and barley, with *F. graminearum* being the dominant species in maize as well. HSI-based DON detection has attracted extensive research attention, yielding a substantial body of work across multiple food matrices and detection scenarios [25,31,32].

Barbedo et al. conducted foundational investigations into DON screening in wheat kernels, demonstrating that while direct quantification of DON concentration using hyperspectral images remained challenging, the relationship between spectral features and DON levels enabled effective classification of contaminated versus safe samples [28]. The study identified specific spectral intervals (1400–1500 nm and 1900–1950 nm) as most informative for DON discrimination, corresponding to water and starch absorption features modified by *Fusarium* infection [28].

Liang et al. conducted a systematic comparison of Vis-NIR and SWIR HSI systems for DON detection in *Fusarium* head blight wheat kernels, revealing that SWIR HSI provided superior classification performance due to its sensitivity to protein and carbohydrate structural changes induced by fungal infection [30]. The study demonstrated that integration of spectral features from both wavelength regions through data fusion strategies enhanced detection accuracy beyond either individual system [10,30].

Femenias and colleagues established a comprehensive research program addressing DON detection standardization, a critical gap often overlooked in laboratory studies. Their work systematically addressed image acquisition standardization [38], single kernel sorting protocols [39], simultaneous DON and ergosterol estimation [40], and *Fusarium* damage evaluation [49]. The standardization of image acquisition methods reduced inter-laboratory variation and improved model transferability across different HSI instruments [38,39]. The simultaneous estimation of DON and ergosterol (a quantitative marker of fungal biomass) provided complementary information distinguishing fungal presence from toxin production [40].

Recent advances have addressed quantitative DON prediction at regulatory threshold levels. Shen et al. achieved rapid, non-destructive quantification of DON in individual wheat kernels using NIR-HSI and chemometrics, developing PLSR models that predicted DON concentrations across the range of 0–12 mg/kg with R^2^ values exceeding 0.85 [32]. Vicens-Sans et al. demonstrated the application of NIR-HSI as a sorting tool for DON reduction in wheat batches, classifying 600 individual kernels according to DON levels and achieving significant reduction in batch-level contamination through selective kernel removal [35].

### 3.4. Ochratoxin A and Fumonisins

OTA, produced by *Penicillium verrucosum* in temperate cereals and *Aspergillus ochraceus* in coffee and dried fruits, primarily affects cereals, coffee, and dried fruits, and has received comparatively less attention in HSI studies than aflatoxins and DON, reflecting differences in global regulatory priority. Senthilkumar et al. conducted systematic investigations of OTA detection in stored barley [42] and wheat [43], demonstrating that NIR-HSI (1000–1600 nm) could detect OTA contamination through spectral changes associated with *Penicillium* fungal growth. In barley, PCA-based analysis identified significant wavelengths at 1310, 1360, and 1480 nm; notably, the 1480 nm peak was unique to OTA-contaminated samples and was not observed in kernels infected by non-OTA-producing *P. verrucosum* strains [42]. In wheat, significant spectral features were detected at 1280, 1300, and 1350 nm, corresponding to modifications in cellular water content and carbohydrate structure during fungal colonization [43]. These findings illustrate that the characteristic wavelengths for OTA detection depend considerably on the food matrix and its specific biochemical composition. The spectral features detected by HSI reflect matrix-specific metabolic changes induced by fungal colonization, including alterations in water content, carbohydrate structure, and protein composition, rather than direct absorption by OTA molecules. Coffee, despite being a major OTA-affected commodity, presents a distinct analytical challenge due to its complex chemical composition, including high levels of chlorogenic acids, caffeine, and Maillard reaction products formed during roasting, which may introduce spectral interferences at different wavelengths. However, HSI studies specifically targeting OTA detection in coffee remain absent from the literature, representing a notable gap that warrants future investigation. For the time being, the spectral regions identified in cereal-based matrices, particularly the 1300–1360 nm region associated with fungal infection and carbohydrate changes, and the 1480 nm band linked to OTA-specific contamination, provide the most relevant reference wavelengths for OTA detection applications.

Fumonisin detection using HSI has predominantly been studied in combination with aflatoxin co-contamination, reflecting the frequent occurrence of these toxins in maize. Kim et al. demonstrated simultaneous detection of aflatoxins and fumonisins in ground maize using multiple HSI systems, revealing that classification models trained on co-contaminated samples achieved higher accuracy than models trained on single-mycotoxin samples, suggesting synergistic spectral effects [3]. The characteristic wavelengths identified for fumonisin detection (1038, 1110, 1393, and 1480 nm) partially overlapped with aflatoxin-related features, complicating the deconvolution of individual mycotoxin contributions in co-contaminated samples [3,17]. Fumonisins are primarily produced by *Fusarium verticillioides* and *Fusarium proliferatum* in maize.

### 3.5. Zearalenone

ZEN is primarily produced by *Fusarium graminearum* and *Fusarium culmorum* in maize, wheat, and barley under temperate and humid conditions, and frequently co-occurs with DON and other type-B trichothecenes. Recent spectroscopic and imaging studies have begun to expand the analytical toolbox for ZEN detection. Liu et al. demonstrated the feasibility of multispectral imaging combined with machine learning for rapid and non-destructive detection of ZEN contamination in maize kernels, achieving satisfactory prediction performance through wavelength selection algorithms [50]. In wheat, Ji et al. applied a competitive swarm optimization near-infrared (CSA-NIR) technique combined with chemometrics algorithms for quantitative determination of ZEN, further confirming the potential of NIR-based spectroscopic methods as a complementary approach to HSI for ZEN analysis [51]. ZEN detection using HSI remains in its early stages, reflecting the compound’s lower regulatory priority compared to aflatoxins and DON. Zhang et al. reported the first application of interpretable deep learning combined with HSI for rapid detection of ZEN and OTA in corn grits, demonstrating that SHAP (SHapley Additive exPlanations) analysis could identify the core wavelengths and spectral mechanisms contributing to model predictions [40]. The interpretable approach revealed specific spectral regions (1380–1420 nm and 1880–1920 nm) associated with ZEN’s molecular structure, providing mechanistic validation of the detection capability [40].

### 3.6. Co-Occurrence of Multiple Mycotoxins

Multi-mycotoxin contamination represents the prevailing reality in food products, yet most HSI studies have focused on single-mycotoxin detection scenarios [2,16]. Large-scale surveys have consistently demonstrated that co-occurrence is the norm rather than the exception. Weaver et al. reported that over 95% of corn grain and corn silage samples collected over a seven-year period in the United States contained two or more mycotoxins, with an average of 8.7 co-occurring mycotoxins per sample [52]. Smith et al. comprehensively reviewed the natural co-occurrence of mycotoxins across major food and feed commodities, identifying frequent combinations such as aflatoxins with fumonisins in maize, deoxynivalenol with zearalenone in wheat and barley, and ochratoxin A with citrinin in cereals, while also highlighting the in vitro combined toxicological effects that may differ substantially from individual mycotoxin toxicity [53]. The simultaneous detection of multiple mycotoxins introduces substantial analytical complexity, as spectral contributions from different mycotoxin–fungal systems may overlap, synergize, or interfere [2,16]. Notably, a growing number of studies have demonstrated the feasibility of HSI-based multi-mycotoxin or multi-analyte detection. Kim et al. simultaneously detected AFB1 and OTA in maize kernels using NIR-HSI (900–1700 nm) combined with CNN, achieving high prediction accuracy for both toxins in a single analytical workflow [3]. Zhang et al. extended this concept to a SWIR-HSI platform (1000–2500 nm) for the concurrent detection of multiple *Fusarium*-derived metabolites in wheat, demonstrating that spectral fingerprints captured in the short-wave infrared region contain sufficient information to resolve co-contaminant signals [19]. Femenias et al. applied NIR-HSI (900–1700 nm) to estimate both DON and ergosterol levels in wheat samples, establishing a dual-analyte model that correlated spectral features with fungal biomass and toxin concentration simultaneously [40]. Most recently, Teixidó-Orries et al. employed Vis-NIR spectroscopy (350–2500 nm) and NIR-HSI for the detection of T-2 and HT-2 toxins in individual oat grains, achieving 93.3% classification accuracy for toxin levels above and below EU regulatory thresholds, thereby demonstrating the potential of vibrational spectroscopy for simultaneous type-A trichothecene monitoring at the single-kernel level [44]. Despite this analytical challenge, advanced machine learning architectures, particularly multi-task learning frameworks and ensemble methods, offer potential for deconvolving multi-mycotoxin spectral signatures and enabling simultaneous quantification [16,26]. However, systematic validation across diverse commodity matrices remains limited. Future research should prioritize the development of multi-analyte HSI methods that reflect realistic contamination scenarios, thereby bridging the gap between laboratory-scale proof-of-concept studies and practical food safety monitoring applications.

## 4. Machine Learning and Deep Learning Integration

The extraction of meaningful information from hyperspectral data cubes is fundamentally a computational challenge. The high dimensionality, strong inter-band collinearity, and inherent noise characteristics of hyperspectral data necessitate sophisticated analytical approaches to identify the subtle spectral patterns associated with mycotoxin contamination. The evolution of computational methods in this field mirrors broader trends in machine learning and artificial intelligence, progressing from classical linear methods through kernel-based approaches to modern deep learning architectures. This section provides a comprehensive evaluation of the machine learning and deep learning algorithms that have been applied to HSI-based mycotoxin detection, examining their respective strengths, limitations, and suitability for different analytical scenarios.

### 4.1. Conventional Machine Learning Algorithms

The HSI–mycotoxin detection pipeline relies fundamentally on computational algorithms to extract classification or regression models from high-dimensional spectral data. Partial least squares regression (PLSR) and PLS-DA are among the most widely used methods, valued for their effectiveness in handling high-dimensional, collinear spectral data [19,20,27,32]. PLSR constructs latent variables that maximize covariance between spectral predictors and mycotoxin response variables, achieving robust performance even when sample sizes are limited relative to spectral dimensionality [27,32]. Support vector machines (SVM) have been extensively validated for mycotoxin classification tasks, particularly in distinguishing contaminated from uncontaminated samples [27,54,55]. SVM constructs optimal hyperplanes in a high-dimensional feature space, offering strong generalization performance for binary and multi-class mycotoxin classification [54]. Random forests (RF) provide robust ensemble classification with inherent feature importance ranking, enabling simultaneous classification and spectral feature identification [17,31].

The comparative performance of conventional ML algorithms varies across application scenarios. PLSR tends to excel in quantitative prediction tasks where the relationship between spectral features and mycotoxin concentration approximates linearity [37,38]. In contrast, SVM and RF demonstrate superior performance in classification tasks with complex, non-linear decision boundaries [31,54,55]. For example, Mansuri et al. systematically compared PLS-DA, ANN (artificial neural network), and 1D-CNN for fungal contamination detection in maize, revealing that 1D-CNN achieved the highest accuracy (94.2%) but required substantially more training data and computational resources than PLS-DA or ANN [31]. Importantly, their study also identified that germ orientation during image acquisition significantly influenced model performance, with dorsal-side scans yielding consistently higher accuracy than ventral-side scans, highlighting the sensitivity of ML models to sample preparation protocols [31].

### 4.2. Deep Learning Architectures

The application of deep learning to HSI-based mycotoxin detection has expanded rapidly, driven by the capacity of neural networks to automatically learn hierarchical feature representations from raw spectral data [20,26,56]. One-dimensional convolutional neural networks (1D-CNN) process spectral vectors as sequential data, extracting local spectral patterns through convolutional filters that identify wavelength intervals most discriminative for mycotoxin detection [26,31,56]. For instance, Soni et al. demonstrated that 1D-CNN outperformed random forest for microbial contaminant quantification when sufficient training data were available, with the architecture automatically learning feature representations without requiring manual wavelength selection [56]. Three-dimensional CNNs (3D-CNN) extend this approach by processing the complete spatial-spectral data cube, simultaneously extracting both spectral and spatial features such as fungal colony morphology and contamination pattern distribution [17,57]. This spatial context information complements spectral data, enabling more robust detection in heterogeneous samples, although 3D-CNN models require substantially larger training datasets and computational resources than 1D-CNN or conventional ML methods [17,26,57]. Transformer architectures represent the frontier of deep learning for HSI analysis. Guo et al. developed a dual-aspect attention spatial-spectral transformer (DAASST) for detecting *Aspergillus flavus* contamination in peanut kernels, demonstrating that self-attention mechanisms could capture long-range spectral dependencies more effectively than convolutional operations [21]. The transformer’s global receptive field enabled integration of spectral information across the full wavelength range, improving detection sensitivity for low-level contamination [21]. Hybrid CNN-transformer models combine local feature extraction with global dependency modeling, offering complementary advantages for complex spectral classification tasks [20,21].

### 4.3. Model Robustness and Transferability

A persistent challenge in HSI-based mycotoxin detection is model transferability across different instruments, sample origins, and contamination conditions. Models trained on data acquired from a specific HSI system with particular experimental parameters frequently suffer performance degradation when applied to data from different instruments or sample populations [27,37,58]. Femenias et al. systematically addressed this challenge by establishing standardized image acquisition protocols and developing calibration transfer methods that maintained model accuracy across different NIR-HSI instruments [27,37].

Transfer learning has emerged as a strategy for addressing model transferability limitations. Deng et al. explored transfer learning approaches for NIR spectroscopy-based mycotoxin detection across different contaminants and grain matrices, demonstrating that pre-trained models could be fine-tuned with limited target-domain data to achieve competitive performance [25]. Zhang et al. combined transfer learning with hyperspectral data for identifying mild *Fusarium* head blight infection severity in wheat, developing a transfer component analysis approach that reduced domain shift between training and testing datasets [59].

Despite these advances, systematic evaluation of model transferability remains insufficient across the HSI–mycotoxin literature. Most studies report performance metrics based on internal validation (cross-validation or hold-out testing within the same dataset) without external validation on independently acquired data [15,16]. This methodological limitation overestimates real-world detection performance and hinders progress toward practical deployment [15,17].

## 5. Spectral Preprocessing and Feature Selection

The quality and interpretability of hyperspectral data are profoundly influenced by preprocessing choices and the selection of informative spectral features. Raw hyperspectral measurements contain not only chemically relevant information but also systematic artifacts arising from light scattering, instrumental noise, baseline drift, and environmental variations. Effective preprocessing is essential to isolate the spectral signals genuinely associated with mycotoxin-related biochemical changes from these confounding sources of variation. Similarly, feature wavelength selection addresses the curse of dimensionality by identifying the most informative spectral bands, enabling parsimonious models that are computationally efficient and mechanistically interpretable. This section examines the principal preprocessing strategies and feature selection methodologies that have been developed and evaluated in the context of HSI-based mycotoxin detection.

### 5.1. Preprocessing Strategies

Hyperspectral data inherently contain various sources of noise and systematic variation that must be addressed before modeling. Scatter effects caused by sample surface heterogeneity, particle size variation, and instrumental drift introduce multiplicative and additive distortions to spectral measurements [16,50]. Standard preprocessing techniques include multiplicative scatter correction (MSC), standard normal variate (SNV), Savitzky–Golay (SG) smoothing and derivative computation, and noise reduction filtering [16,30,50].

MSC and SNV correct for light scattering effects by normalizing spectral intensity, improving the comparability of spectra acquired from samples with different physical properties [16,30]. SG smoothing reduces high-frequency noise while preserving spectral shape, and first or second derivative computation enhances spectral features by eliminating baseline offsets and amplifying subtle spectral differences [16,30]. The choice of preprocessing strategy significantly influences model performance, and systematic comparison of multiple preprocessing combinations is recommended for each application [16,30].

Xu et al. critically evaluated the impact of data quality on HSI assessment results in food applications, emphasizing that preprocessing choices must be aligned with the specific analytical objective and sample characteristics [50]. Overly aggressive preprocessing may remove biologically relevant spectral information, while insufficient preprocessing may retain noise that degrades model performance [50].

### 5.2. Feature Wavelength Selection

The hundreds of spectral bands acquired by HSI systems contain substantial redundancy and noise that can degrade model performance and increase computational requirements [2,16,28]. Feature wavelength selection identifies the most informative spectral intervals, reducing data dimensionality while maintaining classification or prediction accuracy [2,28,51].

Competitive adaptive reweighted sampling (CARS) iteratively selects wavelengths based on regression coefficient magnitude, eliminating redundant and irrelevant variables through an adaptive sampling strategy [30,52]. Successive projections algorithm (SPA) constructs wavelength subsets with minimal collinearity, improving model interpretability [51]. Zhou et al. developed a feature selection approach combining image-based and spectrum-based information for AFB1 classification, identifying characteristic wavelengths that reflected both chemical composition and morphological changes associated with fungal infection [28]. Su et al. applied improved feature variable selection for automated DON determination in barley kernels, combining regression coefficient analysis with iterative optimization to identify optimal wavelength subsets [51].

The mechanistic interpretation of selected wavelengths provides validation of the detection approach. Wavelengths selected for aflatoxin detection (956, 984, 1046, 1350–1450, and 1900–2100 nm) correspond to molecular vibrations associated with fungal metabolic products and seed composition changes [12,27,29]. Wavelengths associated with DON detection (1408, 1904, 2100–2200 nm) reflect protein, starch, and water absorption modifications induced by *Fusarium* infection [28,30]. Wavelength selection for OTA detection (1300, 1350, 1480 nm) corresponds to cellular water and carbohydrate structural changes during *Penicillium* growth [17,42]. This mechanistic alignment between selected wavelengths and known fungal metabolic biochemistry strengthens confidence in the detection validity.

## 6. Complementary Spectroscopic Modalities

**Figure 3:**
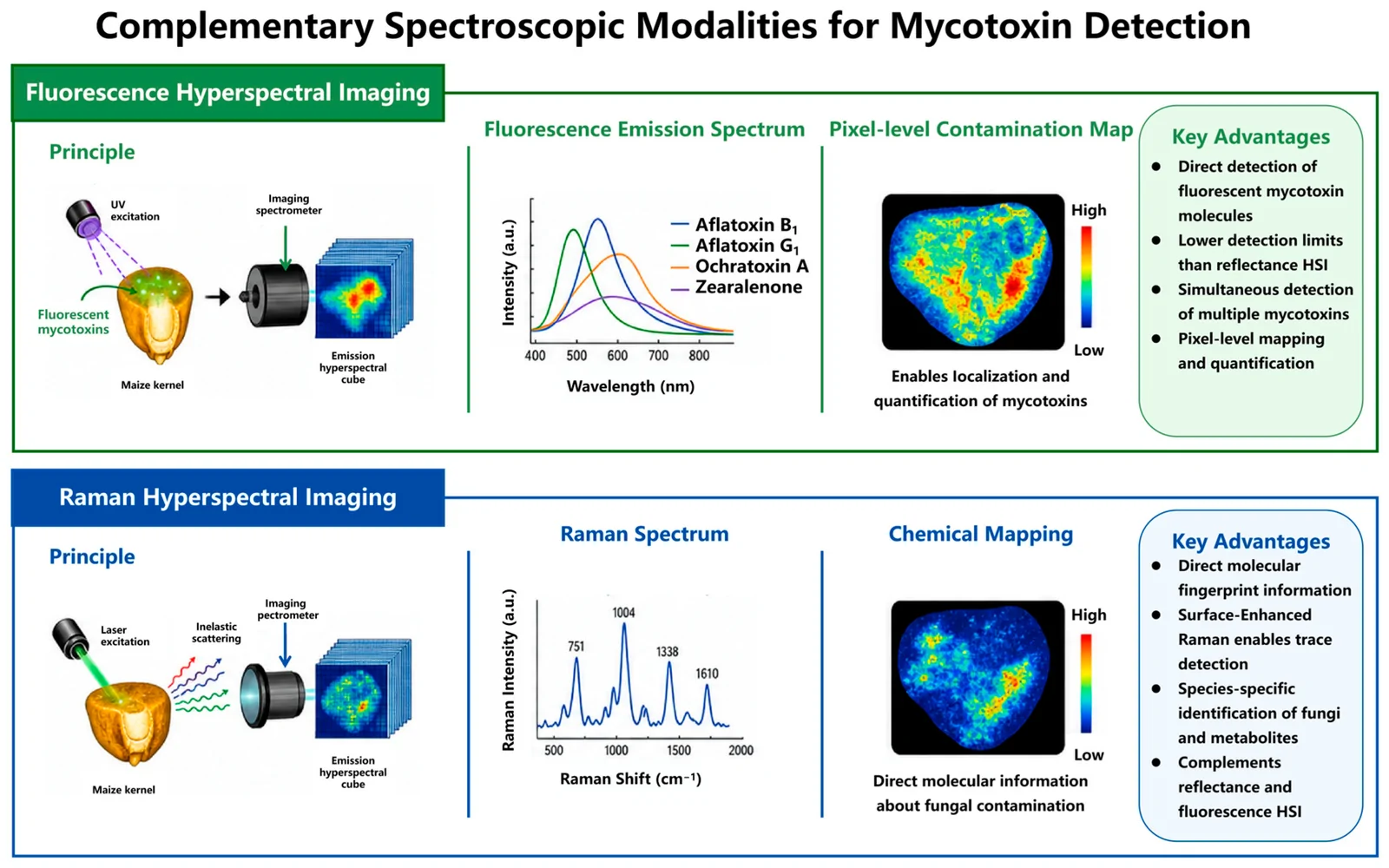
Complementary spectroscopic modalities for mycotoxin detection. Fluorescence HSI exploits the autofluorescence of mycotoxins under UV excitation [22,33], capturing emission spectra and spatial fluorescence distribution for pixel-level contamination mapping [22,48]. It offers lower detection limits than reflectance HSI and enables simultaneous multi-mycotoxin detection [22,60]. Raman HSI provides direct molecular fingerprint information through inelastic light scattering [23,61,62], with Surface-Enhanced Raman Spectroscopy (SERS) enabling trace-level detection [32,61,62]. Data fusion of Raman and HSI information further enhances detection accuracy [23,62]. Together, these modalities complement reflectance HSI by addressing its limitations in detecting non-fluorescent or trace-level mycotoxins.

While reflectance-based HSI has dominated mycotoxin detection research, several complementary spectroscopic modalities offer distinct advantages that address fundamental limitations of the reflectance approach. Fluorescence imaging exploits the inherent photophysical properties of mycotoxins and fungal metabolites, enabling direct molecular detection rather than the indirect inference from matrix changes. Raman spectroscopy provides molecular fingerprint information through inelastic scattering, offering the potential for unambiguous chemical identification. These complementary techniques, individually and in combination with HSI, represent important additions to the analytical toolkit for mycotoxin monitoring. This section reviews the current state and emerging potential of fluorescence hyperspectral imaging and Raman hyperspectral imaging as complementary approaches to reflectance-based HSI for mycotoxin detection. These complementary modalities are illustrated in Figure 3.

### 6.1. Fluorescence HSI

Fluorescence imaging exploits the inherent fluorescence properties of mycotoxins and fungal metabolites, offering detection sensitivity complementary to reflectance-based HSI [21,22]. Aflatoxins exhibit characteristic fluorescence under UV excitation, with BGYF imaging historically serving as a screening method for contaminated corn [21]. Kazemi and Nadimi reviewed the evolution from basic BGYF screening to advanced fluorescence HSI and portable detection systems, demonstrating that fluorescence approaches achieve lower detection limits than reflectance HSI for aflatoxin analysis [22].

Fluorescence HSI captures both emission spectra and spatial fluorescence distribution, enabling pixel-level contamination mapping and quantification [22,23]. Venturini et al. demonstrated multi-mycotoxin detection using fluorescence spectroscopy combined with machine learning, achieving simultaneous identification of multiple co-occurring mycotoxins in maize at concentrations relevant to regulatory limits [53]. The fluorescence approach directly detects mycotoxin molecules rather than indirect matrix changes, addressing a fundamental limitation of reflectance HSI [22,53].

### 6.2. Raman HSI

Raman spectroscopy provides molecular fingerprint information through inelastic light scattering, offering direct detection of mycotoxin molecular structures [54,55,56]. Surface-enhanced Raman spectroscopy (SERS) dramatically amplifies Raman signals through plasmonic enhancement, enabling detection at trace concentrations [18,55]. Long et al. integrated textural and spectral features of Raman HSI for quantitative determination of maize kernel mildew, demonstrating that Raman spectral features provided direct chemical information about fungal contamination that complemented reflectance HSI data [54].

The combination of Raman and HSI information through data fusion strategies represents an emerging approach for enhanced mycotoxin detection [54,56]. Yang et al. identified peanut kernels infected with multiple *A. flavus* fungi using line-scan Raman HSI, demonstrating that Raman spectral signatures of fungal metabolites enabled species-specific identification [56]. Logan et al. reviewed advances in SERS for mycotoxin detection, highlighting the potential of portable SERS systems for field-based screening [55]. The integration of SERS with HSI could combine the spatial mapping capability of imaging with the molecular specificity of Raman spectroscopy, though systematic development of fusion methodologies remains in its early stages [55,56].

## 7. Industrial Implementation and Portable Systems

The translation of HSI-based mycotoxin detection from controlled laboratory environments to practical industrial application represents one of the most significant challenges facing this field. Industrial grain processing operations demand throughput rates, environmental robustness, and system reliability that far exceed the conditions under which most laboratory research is conducted. Simultaneously, the need for accessible mycotoxin screening in resource-limited settings, including smallholder farming operations and developing regions where contamination risks are often highest, has driven the development of portable and low-cost alternatives to conventional laboratory HSI systems. This section examines the current state and future prospects of industrial HSI deployment and portable system development for mycotoxin monitoring, identifying the key technical and practical barriers that must be overcome to realize the transformative potential of this technology.

### 7.1. From Laboratory to Industry

The translation of HSI-based mycotoxin detection from laboratory research to industrial application faces several interconnected challenges [8,17]. Throughput requirements for grain processing facilities (typically thousands of tons per hour) exceed the capacity of conventional push-broom HSI systems [6,17]. The computational demands of real-time spectral analysis require hardware acceleration and optimized algorithms suitable for deployment in production environments [17,24]. Model robustness under variable environmental conditions (temperature, humidity, dust) must be validated to ensure reliable performance outside controlled laboratory settings [27,37,50].

Femenias et al. established standardized NIR-HSI protocols for wheat kernel sorting according to DON levels, demonstrating the feasibility of HSI-based sorting at laboratory scale with potential for industrial scaling [37]. Vicens-Sans et al. evaluated NIR-HSI as a sorting tool for DON reduction in wheat batches, achieving significant contamination reduction through selective kernel removal based on HSI classification [35]. These studies demonstrate the technical feasibility of HSI-based sorting, though scale-up to industrial throughput rates remains to be demonstrated [35].

The integration of IoT sensors with HSI systems enables real-time data collection, remote monitoring, and automated mycotoxin assessment, offering a foundation for industry-scale deployment [16,57]. Mu et al. discussed the integration of AI, big data, and IoT technologies for food safety early warning systems, emphasizing that HSI data could serve as a critical input for real-time contamination monitoring across the food supply chain [57].

### 7.2. Portable and Low-Cost Systems

The development of portable and low-cost HSI systems addresses the need for field-based and resource-limited mycotoxin screening [12,59]. Yao et al. developed a low-cost portable device for detecting and sorting AFB1-contaminated maize kernels, utilizing fluorescence imaging technology adapted for small-scale agricultural operations in developing regions [59]. The device achieved acceptable detection accuracy while maintaining cost levels accessible to smallholder farmers [59].

Handheld spectral devices and portable spectrometers represent an alternative approach to portable mycotoxin detection, trading spatial resolution for portability and ease of use [12,63]. Müller-Maatsch and van Ruth reviewed handheld devices for food authentication, noting that while handheld spectral systems lack the spatial resolution of laboratory HSI systems, they offer advantages for rapid screening in field applications [63]. The development of hybrid approaches, combining portable fluorescence or Raman spectroscopy with targeted laboratory HSI analysis, provides a practical pathway for resource-constrained settings [59,63].

### 7.3. Regulatory Compliance and Pathways to Legal Acceptance

The practical deployment of HSI for mycotoxin monitoring in food ultimately depends on whether the technology can meet the regulatory requirements established by food safety authorities across major jurisdictions worldwide. Current regulatory frameworks impose stringent maximum levels for mycotoxins in food commodities that present significant challenges for HSI-based detection methods. In the European Union, Commission Regulation (EC) No 1881/2006, as subsequently amended by Commission Regulation (EU) 2023/915, establishes maximum levels for aflatoxins in the range of 2–4 μg/kg for aflatoxin B1 and 4–15 μg/kg for total aflatoxins in directly consumed cereals and maize, depending on the food category [64]. In the United States, the Food and Drug Administration (FDA) has established action levels of 20 μg/kg for total aflatoxins in food and 100 μg/kg in animal feed, with lower action levels applying to specific commodities and uses [65]. In China, the national food safety standard GB 2761-2017 specifies maximum permissible levels of aflatoxin B1 at 5–20 μg/kg in various cereal and nut products, with corresponding limits for other mycotoxins including deoxynivalenol (1000 μg/kg in wheat), ochratoxin A (5 μg/kg in cereals), and fumonisin B1 (1000 μg/kg in maize) [66]. While HSI has demonstrated classification accuracies exceeding 90% for distinguishing contaminated from uncontaminated samples in controlled laboratory settings [12,27,29], the technology currently faces fundamental limitations in direct quantification at these regulatory threshold levels, as the indirect detection mechanism captures spectral modifications of the food matrix rather than mycotoxin molecular signatures at trace concentrations [7,9,28]. This regulatory gap suggests that HSI is currently more realistically positioned as a high-throughput screening method rather than a confirmatory analytical technique capable of replacing reference methods such as HPLC or LC-MS/MS.

For HSI-based screening methods to achieve regulatory acceptance, they must satisfy established analytical validation frameworks. The AOAC International provides standardized guidelines for method validation through its Official Methods of Analysis program, which specifies performance parameters including limit of detection (LOD), limit of quantification (LOQ), recovery, repeatability, reproducibility, and probability of detection for screening methods [67]. Similarly, ISO/IEC 17025 [68] accreditation requirements mandate that testing laboratories demonstrate method validation through comprehensive assessment of trueness, precision, measurement uncertainty, selectivity, sensitivity, and robustness. The European Union Reference Laboratories (EURLs) for mycotoxins have established additional performance criteria for analytical methods used in official food control, including requirements for measurement uncertainty, recovery ranges, and specificity that any screening method must satisfy [69]. For HSI to meet these validation requirements, several technology-specific challenges must be addressed: the standardization of spectral acquisition protocols across different instruments and laboratories, the establishment of validated performance metrics specific to imaging-based detection (including spatial resolution effects and pixel-level classification accuracy), and the demonstration of model transferability across diverse sample populations and environmental conditions [38,39]. Notably, AOAC has recently developed Standard Method Performance Requirements (SMPRs) for mycotoxin screening techniques that explicitly accommodate non-conventional analytical approaches, providing a potential pathway for HSI method validation [67].

A pragmatic framework for integrating HSI into mycotoxin monitoring programs is the two-tiered screening strategy. Under this approach, Tier 1 employs HSI as a high-throughput primary screening tool for rapid classification of samples into low-risk (compliant) and potentially non-compliant categories at the point of intake or along the processing line, while Tier 2 directs all suspicious or borderline samples to conventional laboratory-based confirmatory analysis using validated reference methods such as LC-MS/MS. This tiered approach offers several practical advantages: it leverages the non-destructive, rapid, and spatially resolved capabilities of HSI for initial triage of large sample volumes, while maintaining the analytical rigor of reference methods for definitive quantification and regulatory compliance. The two-tiered strategy is legally defensible provided that the screening method demonstrates acceptably low false negative rates (sensitivity approaching 100% at regulatory threshold levels) and that the confirmatory tier satisfies all requirements of the relevant regulatory framework. This approach is conceptually aligned with the screening-confirmatory paradigm already established in EU Commission Regulation (EU) 2023/915 for certain food contaminants and in Codex Alimentarius guidelines for mycotoxin control [35,65]. However, formal validation of HSI-based screening within this tiered framework, including inter-laboratory collaborative studies to establish performance baselines and regulatory acceptance criteria, remains an essential prerequisite for implementation.

Realizing the potential of HSI for regulatory-compliant mycotoxin monitoring will require several foundational developments. First, the development of certified reference materials (CRMs) specifically designed for HSI calibration and validation is essential; unlike conventional analytical methods that rely on chemical standards, HSI-based detection requires matrix-matched reference samples with known levels of mycotoxin contamination that account for the indirect nature of spectral detection through fungal metabolic changes [67,70]. Second, the establishment of standardized hyperspectral datasets with consistent ground-truth measurements, covering diverse mycotoxin–matrix combinations and acquisition conditions, would enable cross-study comparison, model benchmarking, and the development of universally applicable detection algorithms [16,18]. Third, standardized protocols for image acquisition, spectral preprocessing, and model reporting must be developed through inter-laboratory collaborative efforts to ensure reproducibility and comparability of results across different HSI platforms [38,63]. These infrastructure investments, while substantial, represent necessary foundations for transitioning HSI from a promising laboratory technology to a regulatory-accepted tool for food safety monitoring.

## 8. Challenges and Future Perspectives

Despite the substantial progress achieved in HSI-based mycotoxin detection over the past decade, significant challenges remain that must be addressed before this technology can fulfill its potential for routine industrial application. These challenges span fundamental technical limitations, methodological gaps, and strategic priorities for future research. At the same time, emerging developments in artificial intelligence, optical engineering, and data science present unprecedented opportunities to overcome existing barriers and open new frontiers in non-destructive mycotoxin monitoring. This section provides a critical assessment of the fundamental challenges confronting the field, identifies emerging research directions with the greatest potential for impact, and offers strategic recommendations to guide future research and development efforts toward practical implementation.

### 8.1. Fundamental Technical Challenges

The indirect nature of HSI-based mycotoxin detection remains the most fundamental limitation. Since HSI captures spectral modifications of the food matrix rather than direct mycotoxin molecular signatures, detection sensitivity and accuracy depend on the strength of the correlation between observable spectral changes and actual mycotoxin concentration [6,8,25]. This correlation varies substantially across mycotoxin classes, food matrices, and contamination scenarios, limiting the development of universal detection protocols [8,15,25].

A critical methodological distinction that has received insufficient attention in the HSI-mycotoxin literature concerns the nature of sample preparation. Studies in this field can be broadly categorized into two groups: those utilizing naturally contaminated or experimentally infected commodities, where fungi actually grow and produce toxins in situ, and those employing purified mycotoxins artificially spiked into clean matrices. This distinction is particularly consequential for HSI-based detection, given its indirect measurement principle. When a food matrix is spiked with purified mycotoxin, the biochemical and structural modifications that normally accompany fungal colonization, including cell wall degradation, starch hydrolysis, protein breakdown, lipid peroxidation, and tissue microstructure disruption, are entirely absent [7,9,28]. Consequently, spectral signatures from spiked samples primarily reflect the direct absorption features of the toxin molecule superimposed on the background matrix, rather than the composite biological response that characterizes natural contamination. Models trained on spiked samples may therefore achieve apparently strong discrimination or regression performance that does not necessarily translate to naturally contaminated commodities, where the spectral changes are more heterogeneous, spatially variable, and influenced by the dynamic interplay between fungal metabolism and host tissue responses [12,29]. Future studies should prioritize the use of naturally contaminated samples for method development and validation, or at minimum, clearly report the sample preparation approach and explicitly acknowledge the limitations of spiking-based validation when interpreting model performance.

Model transferability across instruments, sample populations, and environmental conditions represents a critical barrier to practical deployment [27,58]. The absence of standardized validation protocols and reference materials for HSI-based mycotoxin detection hinders cross-study comparison and model benchmarking [27,50]. Inter-laboratory collaborative studies with standardized samples and protocols are needed to establish performance baselines and validation criteria for HSI-based detection systems [8,27,50].

Data quality and quantity limitations constrain model development, particularly for deep learning approaches that require large, diverse training datasets [41,50]. Many HSI–mycotoxin studies utilize limited sample sizes (typically fewer than 200 samples), insufficient for training robust deep learning models or comprehensive validation of model robustness [15,41]. The development of shared datasets and benchmarking platforms would accelerate progress in this area [16,17].

The co-occurrence of multiple mycotoxins in the same food commodity presents an additional challenge that is particularly acute for HSI-based detection. Since HSI captures spectral modifications arising from fungal colonization and matrix degradation rather than toxin-specific molecular signatures, the simultaneous presence of multiple fungal–toxin systems introduces substantial spectral overlap and interference. When, for example, *Aspergillus* and *Fusarium* species co-colonize maize kernels, the spectral changes associated with each fungal infection, cell wall degradation, starch hydrolysis, protein breakdown, and lipid peroxidation, occur concurrently and may be difficult to disentangle [7,9,12,28]. Consequently, a model trained to distinguish aflatoxin-contaminated from *Fusarium* toxin-contaminated samples may struggle when both toxin classes are present in the same matrix, as the underlying spectral responses reflect composite biological processes rather than toxin-specific markers [3,17]. Although multi-task learning architectures and spectral deconvolution approaches offer potential for resolving multi-mycotoxin scenarios [16,26], systematic validation of such methods remains limited, and most existing HSI studies have focused on single-mycotoxin detection under controlled laboratory conditions. Addressing this gap will require deliberately designed experiments with co-contaminated samples, standardized reference materials containing multiple mycotoxins at known concentrations, and advanced computational frameworks capable of attributing spectral variations to specific toxin–fungal combinations.

### 8.2. Emerging Research Directions

The application of interpretable deep learning to HSI-based mycotoxin detection addresses the “black box” limitation of conventional neural networks. Zhang et al. demonstrated that SHAP-based interpretability analysis could identify core wavelengths and spectral mechanisms contributing to model predictions for ZEN and OTA detection in corn grits [40]. This interpretability framework provides mechanistic validation of model decisions and builds confidence in detection reliability [40]. The extension of interpretable approaches to other mycotoxin–matrix combinations represents a priority research direction [40,71].

Data augmentation techniques address the training data limitation for deep learning models. Francis et al. reported similar findings for mycotoxin detection in wheat and maize, showing that SVR models with spectral derivative preprocessing and Mixup augmentation achieved improved classification performance [26].

Multi-modal data fusion combines complementary information from multiple analytical techniques to enhance detection capability [9,55]. The fusion of HSI with Raman spectroscopy, fluorescence imaging, or electronic nose data provides comprehensive chemical and spatial characterization that may overcome individual modality limitations [9,54,56]. Guo et al. reviewed spectral data fusion strategies for non-destructive food detection, concluding that data-level, feature-level, and decision-level fusion each offer advantages depending on the application context [9]. The systematic exploration of fusion architectures specific to mycotoxin detection remains an open research opportunity [9,56].

### 8.3. Strategic Recommendations

Based on the critical analysis presented in this review, the following strategic recommendations are proposed for advancing HSI-based mycotoxin detection: (1) Establish standardized validation protocols and representative calibration and validation datasets for cross-study comparison and model benchmarking. Unlike conventional chromatographic methods where reference materials with defined analyte concentrations can be prepared, HSI-based detection captures spectral modifications arising from fungal colonization and matrix degradation, which are strongly influenced by matrix composition, cultivar, moisture content, fungal growth stage, and other biological variables. Reference materials developed for one commodity or cultivar may therefore not provide equivalent spectral characteristics for another. Rather than traditional reference materials, the field would benefit more from representative calibration and validation datasets that deliberately incorporate biological and matrix variability, enabling robust assessment of model generalizability across diverse contamination scenarios. Inter-laboratory collaborative studies should define minimum performance criteria appropriate to the intended application: classification accuracy and misclassification rates for qualitative screening tasks, sensitivity and specificity around regulatory thresholds for compliance testing, and prediction error metrics (RMSEP, bias) for quantitative estimation, recognizing that the concept of ‘limit of detection’ in indirect classification or regression methods differs fundamentally from instrumental LOD in chromatography-based analysis [9,38,63]. (2) Prioritize development of interpretable AI frameworks for HSI-based mycotoxin detection models. Mechanistic validation of model decisions through SHAP analysis, attention visualization, and wavelength importance mapping should become standard practice to build confidence in detection reliability [19,20,21]. (3) Expand investigation of multi-mycotoxin detection scenarios that reflect real-world contamination complexity. Multi-task learning architectures and ensemble methods capable of simultaneous detection of multiple mycotoxins should be systematically developed and validated [3,17,26]. (4) Develop shared benchmarking datasets and open-access model repositories to accelerate research progress and enable reproducibility assessment. The food science community would benefit from standardized datasets covering diverse mycotoxin–matrix combinations with consistent ground-truth measurements [16,18]. (5) Advance snapshot HSI technology for high-speed industrial deployment. Research should focus on improving spatial resolution, spectral coverage, and computational efficiency of snapshot systems to enable real-time contamination screening at industrial throughput rates [18,24].

### 8.4. Roadmap Toward Regulatory-Compliant HSI Deployment

The translation of HSI-based mycotoxin detection from laboratory proof-of-concept to regulatory-accepted industrial technology requires a coordinated, multi-phase research and development strategy. Based on the critical analysis presented in this review, we propose a phased roadmap that identifies key milestones and deliverables for each stage of development, acknowledging that timelines may vary depending on the specific mycotoxin–matrix combination and the regulatory jurisdiction targeted.

Phase 1 (Years 1–3): Foundation Building. The immediate priority is to establish the foundational infrastructure required for rigorous HSI method development and validation. Key milestones for this phase include: (1) creation of standardized, publicly available hyperspectral datasets covering the major mycotoxin–matrix combinations (aflatoxin–maize, DON–wheat, OTA–cereals) with consistent ground-truth measurements obtained through validated reference methods; (2) development of standardized protocols for spectral image acquisition, including specifications for illumination geometry, spatial resolution, spectral range, and environmental conditions, building on the standardization efforts initiated by Femenias et al. [38,39]; (3) establishment of benchmark performance metrics that define minimum acceptable levels for classification accuracy, sensitivity, specificity, and model transferability across instruments and laboratories; and (4) initiation of CRM development programs for key mycotoxin–matrix combinations, produced under ISO 17034 [72] requirements with certified concentration values and stated measurement uncertainties. The expected outcome of this phase is a coordinated research infrastructure that enables reproducible, comparable, and benchmarkable HSI method development across the global research community.

Phase 2 (Years 3–5): Validation and Certification. Building on the foundation established in Phase 1, this phase focuses on formal method validation and regulatory engagement. Key milestones include: (1) execution of multi-laboratory collaborative studies involving at least 10–15 independent laboratories, following the AOAC/IUPAC International Harmonized Protocol for method validation, to establish inter-laboratory reproducibility and define performance baselines for HSI-based screening methods [67]; (2) submission of validated HSI methods for AOAC Performance Tested Methods (PTM) certification or equivalent recognition, with specific emphasis on demonstrating that the methods meet the performance criteria established in Phase 1 across diverse sample populations and acquisition conditions; (3) formal engagement with regulatory agencies (FDA, EFSA, and corresponding national authorities) to define the specific acceptance criteria for HSI as a screening method within the two-tiered framework proposed in Section 7.3, including agreed-upon thresholds for sensitivity, false negative rates, and confirmatory protocols; and (4) development and validation of calibration transfer methods that enable model deployment across different HSI instruments and platforms, addressing the critical barrier of model transferability identified throughout this review [25,38,39]. The expected outcome is formally validated HSI methods with recognized certification status and explicit regulatory guidance on their acceptable use in official food control programs.

Phase 3 (Years 5–8): Industrial Pilot and Deployment. The final phase focuses on demonstrating the practical viability of HSI-based screening at industrial scale and achieving formal regulatory recognition. Key milestones include: (1) deployment of pilot HSI screening systems on industrial grain processing lines, validating throughput capacity (targeting thousands of tons per day), environmental robustness under variable temperature, humidity, and dust conditions, and integration with existing industrial control systems and IoT platforms [18,24,73]; (2) economic feasibility assessment demonstrating that HSI-based screening provides cost advantages over exclusive reliance on laboratory analysis while maintaining equivalent or superior public health protection; (3) implementation of the complete two-tiered screening strategy (HSI screening followed by confirmatory LC-MS/MS analysis) in at least three major grain processing facilities across different geographical regions, with comprehensive documentation of performance metrics, false positive/negative rates, and regulatory compliance outcomes; and (4) pursuit of formal recognition by Codex Alimentarius or individual national regulatory bodies, establishing HSI as an accepted screening methodology within international food safety standards. The expected outcome of this final phase is the establishment of HSI as a validated, economically viable, and regulatorily recognized technology for routine mycotoxin screening in the global food supply chain, representing the culmination of the transition from laboratory research to practical food safety application.

## 9. Conclusions

HSI has established itself as a powerful non-destructive analytical technique for mycotoxin detection in food products, offering the unique capability to simultaneously capture spatial and spectral information across hundreds of wavelengths. This review has critically examined the technological foundations, algorithmic approaches, application achievements, and remaining challenges across the full spectrum of HSI-based mycotoxin research.

Substantial progress has been achieved in several domains. SWIR and Vis-NIR HSI systems have demonstrated classification accuracies exceeding 90% for aflatoxin detection in maize, DON classification in wheat and barley, and OTA detection in stored cereals. The integration of ML and DL algorithms, from conventional PLSR and SVM to advanced 1D-CNN, 3D-CNN, and transformer architectures, has substantially enhanced detection capabilities, particularly for complex multi-mycotoxin scenarios. Spectral preprocessing and feature wavelength selection methodologies have matured, enabling efficient models that maintain high accuracy with reduced computational requirements.

Fundamental limitations persist that constrain practical industrial deployment. The indirect detection mechanism limits quantification accuracy at low concentration levels near regulatory thresholds. Model transferability across instruments, sample origins, and environmental conditions remains insufficiently validated, raising questions about real-world reliability. Training data availability constrains the development of robust deep learning models, particularly for multi-mycotoxin detection scenarios.

The convergence of interpretable deep learning, transfer learning, data fusion, and snapshot imaging technologies presents encouraging pathways for addressing these limitations. Standardized validation protocols, shared benchmarking datasets, and inter-laboratory collaborative studies are needed to establish performance baselines and accelerate progress toward practical implementation. As these foundational elements mature, HSI-based mycotoxin detection systems hold genuine potential for transformative impact on food safety monitoring across the global food supply chain.

## Data Availability

No new data were created or analyzed in this study. Data sharing is not applicable to this article.

## Abbreviations

Abbreviation	Full Name
1D-CNN	1-Dimensional Convolutional Neural Networks
2D-CNN	2-Dimensional Convolutional Neural Networks
3D-CNN	3-Dimensional Convolutional Neural Networks
AFB1	Aflatoxin B1
AFs	Aflatoxins
ANN	Artificial Neural Networks
BGYF	Bright Greenish-Yellow Fluorescence
CARS	Competitive Adaptive Reweighted Sampling
CNN	Convolutional Neural Networks
DL	Deep Learning
DON	Deoxynivalenol
ELISA	Enzyme-Linked Immunosorbent Assay
FBs	Fumonisins
FTIR	Fourier Transform Infrared
HPLC	High-Performance Liquid Chromatography
HSI	Hyperspectral Imaging
LC-MS/MS	Liquid Chromatography-Tandem Mass Spectrometry
ML	Machine Learning
MSC	Multiplicative Scatter Correction
NIR	Near-Infrared
OTA	Ochratoxin A
PCA	Principal Component Analysis
PLS-DA	Partial Least Squares Discriminant Analysis
PLSR	Partial Least Squares Regression
RF	Random Forests
SERS	Surface-Enhanced Raman Spectroscopy
SG	Savitzky–Golay
SHAP	SHapley Additive exPlanations
SNV	Standard Normal Variate
SPA	Successive Projections Algorithm
SVM	Support Vector Machines
SVR	Support Vector Regression
SWIR	Short-Wave Infrared
Vis-NIR	Visible/Near-Infrared
ZEN	Zearalenone
